# “A Bed of Nails”: Professional Musicians’ Accounts of the Experience of Performance Anxiety From a Phenomenological Perspective

**DOI:** 10.3389/fpsyg.2020.605422

**Published:** 2020-11-12

**Authors:** Ioulia Papageorgi, Graham F. Welch

**Affiliations:** ^1^Department of Social Sciences, School of Humanities and Social Sciences, University of Nicosia, Nicosia, Cyprus; ^2^Department of Culture, Communication and Media, Institute of Education, University College London, London, United Kingdom

**Keywords:** music performance anxiety, professional musicians, Interpretative Phenomenological Analysis, semi-structured interviews, lived experience

## Abstract

Most investigations of musical performance anxiety have employed quantitative methodologies. Whereas such methodologies can provide useful insights into the measurable aspects of the experience in a larger group of participants, the complexity, subtlety and individuality of the emotional experience and the importance of the individual’s interpretation of it are often overlooked. This study employed a phenomenological approach to investigate the lived, subjective experience of performance anxiety, as described in professional musicians’ narratives. Semi-structured interviews with four professional musicians (two males, two females) specializing in Western classical and jazz music genres were conducted and analyzed using Interpretative Phenomenological Analysis (IPA). The analysis revealed the presence of four overarching themes: (1) Intensity of performance anxiety experience, (2) perceived effects, (3) development of coping strategies, and (4) achieving release from anxiety. Findings suggest that the lived experience of performance anxiety is multifaceted, characterized by a physical and a psychological dimension. Interpretative Phenomenological Analysis is a useful research tool that can facilitate our understanding of the subjective experience of performance anxiety (how it is felt and understood at an individual level) and can thus be useful in the development of tailor-made intervention programs for musicians.

## Introduction

Debussy once wrote that “the attraction of the virtuoso for the public is very like that of circus for the crowd. There is always a hope that something dangerous may happen” (Debussy, 1962, p. 22). This statement captures the essence of music performance anxiety (MPA) – the fear of public failure that, for many performers, is associated with anxiety. Whereas musicians report high levels of job satisfaction, they are also likely to report mental illness (Brodsky, 1996). Few activities in life can generate tension and anxiety as rapidly and thoroughly as the performance of a musical instrument in a public context (Havas, 1995). Plaut (1990, p. 59) wrote that “Nothing is more devastating to a performing artist than not having the chance to be on stage and, as the pervasiveness of performance anxiety attests, nothing is more threatening than having that chance.” This happens when anxiety and fear take away the pleasure, and performers are overwhelmed by feelings of apprehension, tension, and distress as a result of MPA.

Music performance is an activity that is both physically and psychologically demanding (Papageorgi and Kopiez, 2018). The high levels of technical skills required of professional musicians place excessive demands in musical training and performance. These can interfere with the well-being and health of musicians (Matei and Ginsborg, 2017). The quality of performance is influenced by the performer’s level of expertise and adequacy of preparation, but can be affected by psychological factors, such as self-perceptions, self-efficacy beliefs, and the experience of performance anxiety (Papageorgi et al., 2013). MPA is a complex phenomenon that has gained research attention in recent decades, due to its high prevalence in musicians and the negative impacts it can have on their career and quality of life (Burin and Osorio, 2017). Many studies report that female adult musicians experience higher levels of performance anxiety (Fishbein et al., 1988; Wesner et al., 1990; Papageorgi, 2008; Iusca and Dafinoiu, 2012; Papageorgi et al., 2013; Coskun-Senturk and Cırakoglu, 2018; Gonzalez et al., 2018).

Music performance anxiety is prevalent in professional musicians, with literature reporting varying levels of prevalence, depending on the focus group and methodology. Studies with adult orchestral and choral musicians, for example, have reported a prevalence of between 15 and 70% (Schulz, 1981; Gustafson and Rawson, 1983; Fishbein et al., 1988; Marchant-Haycox and Wilson, 1992; Steptoe, 2001; Brugués, 2009; Ryan and Andrews, 2009; Medeiros Barbar et al., 2014). Recently, a systematic review of MPA in professional musicians concluded similarly that prevalence rates range between 16.5 and 60% (Fernholz et al., 2019).

Contextual factors, such as the characteristics of a specific performance setting, solo or group performance and the musical genre, contribute in determining how a performer will perceive and interpret the performance conditions (Papageorgi, 2020). Situational factors can influence musicians’ ability to cope with the demands of performance – for example, it has been suggested that the formality of the Western classical context may contribute in evoking additional pressure and elevating anxiety levels (Papageorgi et al., 2013). Musicians from the Western classical genre have been found to experience higher levels of MPA compared to popular and jazz musicians (Papageorgi et al., 2013; Perdomo-Guevara, 2014; Nusseck et al., 2015). Furthermore, there is evidence to suggest that the type of performance can influence the presence of MPA. Perception of high levels of personal exposure (such as in solo performances) and direct or indirect assessment (such as in the presence of an audience or examiners, an audition or competition in front of a jury) appear to elevate MPA levels (Papageorgi et al., 2010b). Variations in MPA level can also be evident in relation to the timeline of performance. In a longitudinal study, Hildebrandt et al. (2012) found that first year undergraduate music students reported comparable levels of MPA on the day of a concert and during the concert at the beginning of the academic year, but reported significantly higher levels of MPA during the concert at the end of the academic year. Papageorgi et al. (2013) also found variations in reported MPA levels 1 h, immediately before and during solo and group performances of undergraduate and professional musicians, with anxiety peaking immediately before and decreasing during the performance.

Music performance anxiety can have significant negative effects (i.e., maladaptive MPA), as evidenced through physiological, mental (cognitive and emotional) and behavioral symptoms reported in the literature (Gabrielsson, 1999; Steptoe, 2001; Yoshie et al., 2009; Papageorgi, 2014; Burin and Osorio, 2017). Nevertheless, for some performers, MPA has facilitating effects (i.e., adaptive MPA) such as improving physical readiness for the demands of performance, increasing motivation to succeed, aiding concentration, and enhancing the overall quality of performance (Papageorgi, 2008, 2009, 2014; Papageorgi et al., 2013; Larrouy-Maestri and Morsomme, 2014). Evidence from these studies suggest that the facilitating effects are more likely to be observed in more experienced performers, such as professional musicians.

### The Study of MPA

The majority of research in the field has maintained a clinical approach to the study of performance anxiety, focusing on its etiology, prevalence and treatment (see, for example, the systematic reviews of Burin and Osorio, 2017; Fernholz et al., 2019). Most published studies with adult musicians have employed quantitative methodologies, for example: intervention studies and experimental research (e.g., James and Savage, 1984; Gates et al., 1985; Gates and Montalbo, 1987; Nagel et al., 1989; Montello et al., 1990; Brotons, 1994; Brodsky and Sloboda, 1997; Chang et al., 2003; Kim, 2005, 2008. Khalsa and Cope, 2006; Khalsa et al., 2009; Stern et al., 2012; Wells et al., 2012; Bissonnette et al., 2015; Spahn et al., 2016; Juncos et al., 2017) and psychometric testing (e.g., Steptoe and Fidler, 1987; Kenny et al., 2014; Medeiros Barbar et al., 2014; Nielsen et al., 2018). Physiological measurements of MPA (e.g., Abel and Larkin, 1990; Brotons, 1994; Yoshie et al., 2009; Wells et al., 2012; Kenny et al., 2013; Studer et al., 2014; van Fenema et al., 2017; Gomez et al., 2018) have also been used as indicators of physiological arousal resulting from activation of the sympathetic division of the Autonomic Nervous System (ANS), for example heart rate, skin conductance, blood pressure, hormone levels, muscle tension and respiration (Studer et al., 2014).

Quantitative studies have been successful in unraveling the risk factors, prevalence rates, symptomatology and effective treatments of MPA, thus furthering our understanding of the condition. In order to reach convincing conclusions and be able to generalize findings to a population of musicians, the methodological approach quantified the experience of performance anxiety and used inferential statistical testing to analyze obtained data. Limitations of such studies, arising from heterogeneity in the assessment of MPA and methodological weaknesses identified in studies evaluating treatments of MPA (Kenny, 2005), call for the necessity of conducting further research and of adopting a perspective that will allow hearing the actual voice of those experiencing MPA in order to enrich our understanding of the phenomenon. One drawback of quantitative methodologies is that they may not capture the complexity, subtlety and individuality of the emotional experience of MPA and the importance of the individual’s interpretation of it. Idiographic approaches, such as phenomenological psychology (Langdridge, 2007) can be valuable in promoting a more in-depth understanding of MPA at an individual level. Phenomenological psychologists focus on the experience and believe that the study of experience can provide valuable insights into human life (Eatough and Smith, 2017). One approach of phenomenological psychology that has been used in the study of emotional phenomena is Interpretative Phenomenological Analysis (IPA).

### Interpretative Phenomenological Analysis

The focus of IPA is the detailed examination of personal lived experience; its key features include experience, idiography and interpretation (Eatough and Smith, 2017). Information is typically collected through semi-structured case study (or focus group) interviews (Smith et al., 2009). An in-depth account and analysis of each case is made before proceeding to search for patterns of convergence and divergence across cases (Eatough and Smith, 2017). Smith (2011a, b) outlined the key characteristics that constitute good quality IPA: A sustained focus on a particular aspect of experience, rich experiential data, assessment of the thematic structure of data through the assessment of prevalence, careful elaboration of themes and detailed interpretative engagement with the material.

### Phenomenology and IPA in the Study of Emotional Phenomena

Solomon (2006) wrote about the phenomenology of emotions and how such an approach can capture the complexity and richness of our emotional lives. IPA, by employing the ideas of hermeneutic phenomenology, perceives individuals to be continuously emotionally engaged with their world and to interpret their experiences through their unique historical and social context (Gill, 2015). An IPA approach aims to examine and understand how individuals interpret and understand their experiences. Gill (2015) developed five recommendations for the investigation of emotions through the lens of IPA:

(1) Intentionality: Research questions that focus on experience
(2) Dasein: An idiographic approach to sampling
(3) The life-world: Collecting data that reflect the participant’s perspective
(4) Hermeneutic cycle: Iterative data analysis
(5) Double hermeneutic: Reflexively evaluating the research.

The usefulness of employing a phenomenological approach to the study of emotional phenomena has been illustrated by Eatough and Smith (2006), who looked at anger. IPA of two semi-structured interviews with a female participant indicated that anger was an intensely felt emotion, which was transformative as it brought a different person into being. The body was described as central to the experience of anger. Gyllensten et al. (2005) employed a similar methodology to investigate perceptions of work-related stress; their semi-structured interviews with seven participants identified resistance toward receiving counseling for stress as one of the main emerging themes. These studies suggest that phenomenological psychology can be useful in understanding emotional experiences.

More recently, Denovan and Macaskill (2013) investigated stress and coping in first-year undergraduates. Semi-structured interviews with 10 first-year United Kingdom undergraduates that focused on the transition and adjustment to university revealed five main themes: all the change (subthemes of independent living, homesickness, differences between post-compulsory education and university); expectations of university; academic focus (subthemes of self-discipline, motivation, learning from experience); support network (subthemes of establishing a support network, support for coping with problems); and difficulties (subthemes of difficulties experienced with housemates, finances and employment, and academic difficulties).

### IPA in the Study of Music Performance

Clark et al. (2007) employed a phenomenological approach to the study of musical performance. Analysis of 13 semi-structured interviews using IPA indicated that musicians connected successful performances with feelings of sufficient preparation and positive mindsets, and considered these to present a high but attainable level of challenge. Less successful performances were related to a sense of inadequate preparation, negative mental outlooks, frustration, and lack of enjoyment during performance. Burland and Davidson (2002) conducted semi-structured interviews with 18 young adults who had attended a specialist music school for “talented” child and adolescent musicians 8 years previously. The research focus was on understanding the reasons for following a professional career or not. Using semi-structured interviews and IPA, they identified several themes of key importance in determining a successful transition from training to professional life, with the development of coping strategies to deal with the demands of performance being a key theme.

### IPA in the Study of Music Performance Anxiety (MPA)

With regards to the experience of MPA, only a handful of studies have employed a phenomenological approach through the use of IPA. Robinson and Nigbur (2018) conducted semi-structured interviews with four undergraduate classical voice students and reported three themes: The psychological impact of the audience, issues of trust, and musical identity. Cupido (2018) investigated MPA, perfectionism and its manifestation in the lived experiences of singer-teachers. Participants reported the main triggers of MPA to be self-induced pressure and concerns over their voices and success. Self-doubt and singer-teachers’ desire for approval, reassurance, and acceptance from colleagues and students aggravated feelings of MPA. Bober (2019) investigated MPA in popular musicians and identified seven themes emerging from the data: Nuanced musical identity, experiencing memories of MPA, preoccupation with the audience and other musicians, self-doubt, symptoms of MPA, coping strategies, and encountering situational factors. When the themes were analyzed for a second time through the lens of universal existential structures, themes of relationship to self (musical identity and thoughts toward self), relationship to others (audience and other musicians), bodyhood (symptoms of MPA experienced as unwelcome and difficult to control changes in the body), temporality (subjective experience of time during experiences of MPA), causality (cause of MPA was attributed to self), and spatiality (experiences of MPA in relation to space, closeness and distance) emerged.

### The Current Study

The review of the literature suggests that IPA can facilitate in-depth understanding of the lived experience of emotional phenomena. For performing musicians, one of the key emotions likely to be experienced as a result of the demands of the profession is MPA. The current study was designed on the basis of phenomenological psychology principles, which focuses on human experience, is concerned with meaning and how it arises in experience, focuses on a description of the individual lived experience and recognizes that experience must be understood in context (Langdridge, 2007). A particular advantage of phenomenological psychology in the study of emotional phenomena is the centrality of the notion of embodiment. By focusing on how bodies are experienced at a subjective and intersubjective level (Finlay and Langdridge, 2007), it is proposed that the study of MPA can be further enriched by focusing on the subjective body as lived and experienced in order to understand how MPA is manifested at an individual level. The overall aim of the study was to understand the subjective lived experience of MPA in professional musicians.

## Materials and Methods

### Participants

Professional musicians known to the research team through professional networks (e.g., orchestras, music organizations) were approached with information about the study and those musicians who expressed interest to take part were asked to sign a consent form prior to any data collection. Data reported in this paper were part of a 2-year comparative study (Investigating Musical Performance: Comparative Studies in Advanced Musical Learning; funded by the ESRC). It was devised to investigate how musicians across different musical genres deepen and develop their learning about performance in undergraduate, postgraduate and wider music community contexts, as evidenced by the diversity of the working fields of the participants. Four professional musicians (2 male and 2 female)^1^ took part in the current study. All had begun their engagement with music at an early age and came from musical families or families with interest in music. Tom specialized in classical music and was in his mid-fifties. Robert specialized in jazz and was in his early forties. Kelly was a female jazz musician in her mid-thirties. Emma was a classical musician in her early twenties.

### Interview Schedule and Procedure

Data were collected through semi-structured interviews. They can produce rich data and allow for important areas and interesting topics to surface through the discussion to be addressed, and the interview schedule is only used as a guide (Smith and Osborn, 2015). A short pilot interview preceded the main data collection, resulting in minor modifications to the original interview schedule. During the interviews, participants were asked to introduce themselves, talk about their musical history, and then to recall particularly positive and negative experiences as performers. The discussion then explored whether performance anxiety has been an issue for them, and—if so—how MPA feels, and whether its experience has changed over time. The interviews concluded with a discussion of any personal strategies employed to cope with the demands of performance and to reduce any feelings of performance anxiety as understood by each participant. The interviews lasted 45 min – 1 h each, they were audio-recorded using a digital voice-recorder and were transcribed verbatim.

### Ethical Considerations

Ethical approval for the study was obtained from the academic institution in line with BPS guidelines. Participants were informed of the focus of the study, signed a consent form and were debriefed after the completion of each interview. Participants were volunteers and informed of their right to withdraw at any point. Permission to audio-record the interviews was requested and participants were assured that the data would be confidential. Participants’ identity was protected by using pseudonyms and omitting any details that may have rendered them identifiable.

### Data Analysis

Interviews were transcribed verbatim and analyzed using IPA (Smith and Osborn, 2015). Through the epoché, any *a priori* beliefs regarding the experience of performance anxiety were suspended as much as possible. The analytic procedure followed IPA guidelines and involved the steps shown in Table 1 (Smith and Osborn, 2015; Eatough and Smith, 2017).

**TABLE 1:** The analytic procedure.

Analytic procedure employing Interpretative Phenomenological Analysis	
(1)	Reading the first interview transcript several times and make initial notes of interesting points in the margin.
(2)	Re-reading the transcript and transforming initial notes and ideas into more specific themes or phrases
(3)	Reducing the data further by establishing connections between the preliminary themes and clustering them appropriately.
(4)	Giving descriptive titles to the overarching (higher order) themes to capture the conceptual nature of the themes
(5)	Repeating steps 1–4 for the all interview transcripts
(6)	Identifying similarities and differences in the themes derived across the interviews
(7)	Organizing themes and sub-themes from the interviews in a table
(8)	Selecting representative examples (quotations) that capture the essence of each theme and sub-theme

The transcripts and themes were analyzed again with the structures (essential features) of the lifeworld in mind (temporality, spatiality, embodiment, intersubjectivity) (Moustakas, 1994; Eatough and Smith, 2017) to identify if any of these were present in the accounts of participating musicians.

The identified themes were the result of meticulous coding procedure according to IPA guidelines, and these themes were agreed upon by both authors in the reading and re-reading of the narratives. In order to ensure the validity of the data analysis interpretation, shared analysis of data following the recommendations of Rodham et al. (2015) was conducted. Using Rodham et al.(2015, p. 68) as guidance, it is acknowledged that the IPA researcher adopts two positions, the insider perspective and the researcher perspective. The process of reading, re-reading and sharing ensured that the interpretation matched the information being provided in the participants’ narratives, and this procedure safeguarded the trustworthiness and validity of the analysis. Authors also agreed that the selected quotes in the paper were representative of the themes that emerged across the four participants.

## Results

### Emerging Themes

The analysis of the four interviews revealed the presence of four overarching themes, each consisting of a number of sub-themes. These are illustrated in Table 2.

**TABLE 2:** Emerging themes and sub-themes from the interviews^2^.

List of Themes and Sub-Themes	Occurrence
**(1) Intensity of performance anxiety experience**	
1a. Physical dimension	*Tom, Robert*
1b. Psychological dimension	*Tom, Robert, Kelly, Emma*
**(2) Perceived effects**	
2a. Quality of playing	*Tom, Robert, Kelly Emma*
– Negative	
– Positive	
2b. Perceptions of Self	*Tom, Robert, Kelly*
**(3) Development of coping strategies**	
3a. Problem focused	*Tom, Robert, Kelly, Emma*
3b. Emotion focused	*Robert, Kelly, Emma*
**(4) Achieving release from anxiety**	
4a. Reaching professional maturity	*Tom, Robert, Kelly*
4b. Pragmatism	*Robert, Emma*
4c. Supportive peer community	*Tom, Robert, Emma*

*^2^Please note that not all participants may have touched upon a particular theme during their interview. Table shows which participants mentioned each theme.*

### Theme 1: Intensity of Performance Anxiety Experience

Participants described performance anxiety as an intense experience that had a profound effect on them. The lived experience of performance anxiety was described in terms of both a physical and a psychological dimension.

#### Physical dimension of MPA

For Tom, performance anxiety had a strong physical dimension, as he described it in terms of bodily sensations. The experience was compared to “a bed of nails” and was described as painful:

Erm, oh God, lots, lots and lots of negatives (…) And usually it’s been to do with control of my nerves (…). Oh, that’s just so hard! That’s a bed of nails, really is. And that happens, oh, too many times (Tom).

That’s when it really was troublesome, you know (…) Painful really (…) And I suffered, you know it’s definitely something that you endure and it’s a painful part of your life (…) (Tom).

For Robert, the experience had a completely different physical dimension, as he wanted to run away from it:

(…) you’re standing in front of a room full of people and you want to kind of run away (Robert).

#### Psychological dimension of MPA

Robert talked about the intensity of the performance anxiety experience in relation to a psychological dimension, by making reference to how humiliating it was and how it had remained prominently in his memory:

(…) it was kind of humiliating because, you know, it’s that not being quite ready for the live. You can practise in your bedroom and you can get your fingers doing it, but, you know, you got a 20-piece band behind you and it doesn’t… the band don’t stop to let you catch up or whatever… so yeah, I’ve got quite vivid memories of making a mess of it (…) (Robert).

He also talked about how performance anxiety was related to low confidence and the pressure of having to compete with other musicians:

I think anxiety was an issue when I would go to these jam sessions or there would be a jazz festival and there would be all these great musicians and go “oh, they’re great! What am I doing? I can never play like that, therefore I’m rubbish” (Robert).

Kelly spoke about how seeing exceptional musicians playing reduced her confidence:

I suddenly mentally decided that I wasn’t very good and so I didn’t play very well (Kelly).

Emma felt angry with herself after a disastrous performance:

… I was really cross with myself (Emma).

#### Theme 2: Perceived Effects

Participants described in detail the negative effects that performance anxiety had on the quality of their playing, as well as on their self-perception. However, one participant also discussed positive elements.

##### Quality of playing

Tom spoke intensely about how performance anxiety impeded the quality of his performance, prevented him from playing at the standard that he knew he was capable of, and held him back from reaching his full potential:

(…) usually it’s been to do with control of my nerves, control, you know, not doing what I know I am capable of doing when the moment came to it. Not achieving the standard I know I could, maybe nobody else would have noticed, but I noticed (…) (Tom).

The thing that’s held me back as a performer, from perhaps achieving everything that I could have done, is performance anxiety. And you know, it’s just been an issue. And I’ve always had to cope with it, all my life. It’s always been an issue (Tom).

The stresses of performance and how these interfered with the performance was also mentioned by Robert:

And also because you are promoting yourself, then there can be stresses that get in the way of the playing, you know, “oh, is the audience gonna come” or “if is somebody in a bad mood then how’s that gonna affect”… and these things can sometimes get in the way (Robert).

Kelly found that seeing highly competent musicians playing in the same venue increased her anxiety and affected her playing:

And as a result, my playing completely went, didn’t manage to sustain it at all (Kelly).

Emma felt that anxiety prohibited her from enjoying the performance and from interpreting the music and communicating with the audience:

It was ok from a technical point of view, but from a performance point of view and from a communication point of view, it wasn’t at all (…) I was overly nervous and I think it did… it did stop me from enjoying it (Emma).

Emma also spoke about a reconceptualization of MPA into its positive effects, signifying excitement and motivation:

… trying to think of it in terms of not being anxious, but more being excited. Erm… I’m trying to think that that’s a good thing because it shows that I want to perform well and I want to, you know, give the audience a good performance and I’m trying to turn it round to thinking of it like that.

##### Perceptions of self

Participants also spoke about how performance anxiety made them feel negatively about themselves. Tom often felt disappointed:

Once again within my professional playing life I have often come out and been terribly disappointed with my playing (Tom).

Robert described how “making a mess of it” produced negative feelings:

But I’d go away and practise and then try and play like [great musicians] and then make a mess of it and then feel bad. So, yeah, I spent a number of years in the fringes of that (Robert).

For Kelly, the feelings revolved around being a mother and pregnant at the time, and the internal struggle arising from working professionally while raising a family:

I remember one time I was playing piano at a jam session and I was part of the house band and I was very heavily pregnant with my second son, and this young guy came along to play and he sat in on the piano and he was fantastic and I just suddenly, my confidence went and I thought “what am I doing here” you know? “I should be at home nest building. Who am I kidding, going out and trying to gig?” (Kelly).

#### Theme 3: Development of Coping Strategies

Participants spoke about having devised strategies to cope with the demands of performance and reduce performance anxiety. Some strategies were of a practical nature and focused on dealing with the problem at hand (problem focused), whilst other strategies focused on alleviating the negative emotions accompanying performance anxiety (emotion focused).

##### Problem focused strategies

Tom spoke about his experiences of anxiety becoming less problematic by learning effective strategies to cope:

I suppose it did get better because I learned techniques to deal with it, strategies to deal with it… (Tom).

Practising was a key feature in musicians’ coping strategies. Tom tried to practise as much as possible, and stressed the importance of developing metacognitive skills to understand himself as a learner in order to be able to identify areas of concern:

(…) I practise as much as I can, as time will allow. I think I’m better at practising than I used to be when I was younger, because I understand much more about what is needed and what’s involved and I know myself and I know that’s likely to give me trouble. So, I can cut corners (…) (Tom).

Robert also felt that practising was an integral component of a successful preparation for performance:

(…) we’re having conventional band rehearsals…erm. and I’ll also prepare myself with my own personal practice schedule. So if the performance has technical requirements (…) I’ll have to invest time to make sure that I can meet those technical demands, but, at the same time, stay focused on the wider sort of creative improvisatory element (Robert).

Tom and Robert felt that exercising and keeping fit was helpful:

I try to keep myself active and fit, I mean, in the sense that I don’t do any formal exercise, but I do a lot of walking and, you know, that sort of thing… because that’s terribly important when you’re a wind player (Tom).

Occasionally, I do some exercises, physical exercises (…) try to make yourself breathe a bit more, stretch your body out (…) (Robert).

Kelly sought specialized help to increase her confidence levels and start performing again:

I went along to see an NLP [neuro-linguistic programming] practitioner a while ago when I was having some difficulties with… with confidence generally… (…) I mean, I think it was just a therapeutic thing, but it also helped me get through that problem in music so I felt okay about doing it again (Kelly).

Emma focused on practising and internalizing the music in the weeks leading up to the performance:

… in the weeks leading up to it I’ll just do loads and loads of practice, obviously and erm… just… the more, kind of, looking at my music I do and, kind of, listening to it and going through it and… I do a lot of internalizing of it (Emma).

Right before the performance Emma focused on relaxation techniques:

I’ll just do, like, breathing exercises and, kind of, shoulder… you know, things to kind of relax me (Emma).

##### Emotion focused strategies

Robert in particular spoke about strategies that aimed at alleviating the emotional experience of anxiety, such as avoiding performances:

I guess one of my strategies was to hide (…) Secretly I would want probably to do it, I would want to go up there and play really well, but I didn’t have the confidence and so I would avoid it (Robert).

He also spoke about accepting who he is as a musician and maintaining a realistic outlook to performance:

(…) I feel the need less to emulate the past masters… feel less of a requirement to play like other people. I kind of accepted who I am as a musician and that I’m never gonna be the fastest saxophonist, I’m never gonna be able to play all the changes (…) when you accept that, but at the same time, still say “I still want to pay attention to technical demands, but I’m gonna accept that this is what I’m doing,” then there’s a huge weight off your shoulders (Robert).

Kelly, after receiving specialized help, was able to use past positive experiences to prepare psychologically for a performance:

… but as a result of doing that [NLP], I got the idea of being able to focus on past experiences that have been very positive. So the gig I told you about, where we were playing in xxx with my own band, sometimes if I want kind of psych myself up I will just remember… I can quite remember how I felt go on the stage and see lots of people and feel to it was my thing and feel very proud and I’ve got quite a good memory of that and so I can use that to help me (Kelly).

Emma tried to focus her attention to music itself, the enjoyment of the performance and communication with the audience:

I’m trying to kind of think in my head about ‘this is what I’ve chosen to do, this is what I love doing, I’m just going to go out there and I’m going to really enjoy it and I’m just gonna… you know, let the audience see the music, hear the music and not show anything of my insecurities, or anything like that (Emma).

### Theme 4: Achieving Release From Anxiety

Overcoming performance anxiety was a crucial moment in the career of each participant. Their comments suggested that this was achieved through reaching professional maturity, becoming more pragmatic and through peer support.

#### Reaching professional maturity

All participants felt that successful coping and overcoming anxiety came with time and was mediated by reaching professional maturity. This was particularly prominent in Robert’s account:

I think with experience you become less tied to any one particular [performance]. You don’t wanna throw the saxophone away because of one bad gig and, at the same time, you’re not gonna play one great gig and suddenly realize this is going to change my life, in the way in which your formative experiences can (Robert).

Tom spoke about becoming more in control of his nerves with age, and sensing great relief when he finally retired from professional performance work:

I suppose it got better as I got older (…) I must say, having stopped full-time professional playing, that’s something I really have not… I mean, I’ve just thrown that off! It’s been wonderful release! (Tom).

Kelly realized that as she matured in age, she also matured professionally and stopped judging herself through making comparisons with other musicians:

I think I was much more concerned with comparing myself when I was younger. I think now I’m probably a bit kinder to myself… not… you know, not completely, but better that I was. I used to spend a lot of time agonizing about who was better than me and kind of saying to people “yeah, but I’ve only been doing it for 6 months!” or, you know, kind of trying to prove a point. And I’m sure I do some of that now, but it doesn’t seem like quite such a big deal, which is a real relief (Kelly).

#### Pragmatism

The importance of maintaining a realistic outlook to performance in reducing some of the pressure was particularly prominent in Robert’s account:

By accepting who I am and also seeing the ludicrousness of that situation as well (…), unless you conform to very narrow criteria rhythmically, harmonically, socially as well, you’ve got to be able to play the game socially. It’s not gonna work for you and, I think, accepting that and being able to see the jam session for that was a kind of… a realization (Robert).

Emma felt that she had an obligation toward the audience to “deliver the goods”:

I’m just going to, kind of, think about it from the audience’s point of view rather than my own, really. And I’m just there to deliver the goods, as it were. (…) as soon as it’s time to go on then, you just, kind of, leave everything behind and you just… you just go with it, don’t you? You just, kind of, start and carry on and that’s it, you just get on with it, really (Emma).

#### Supportive peer community

Feeling part of a supportive community of peers and making a contribution to the success of the group played a significant role in making performance a positive experience. Communication between co-performers was also considered very important.

Tom’s account focused on the satisfaction arising from making a contribution to the success of a performance:

That’s the most common musical experience that I’ve had in my life, which has been positive, which is that I’ve come out and thought “I did a good job as a member of the team and it came off, you know, and I’m really pleased with that result.” You know, and that gave me satisfaction, tremendous satisfaction (Tom).

Robert’s account focused on the communication between musicians on stage and reaching what may be described as a state of “flow”:

(…) I was involved in a very sort of intimate communication with him. There was 25 of us on stage, but I was very switched on to what he was playing and I felt that we had this real meeting point. And it was such a thrill and I got a real sort of spine tingling, sort of feeling out of playing with xxx again (…) (Robert).

Emma found that talking through things with fellow musicians increased her confidence and helped her understand herself:

Talking through it with other musicians and people who do different things, yeah… That’s helped. The way I look at things and erm… from a confidence point of view and that kind of thing, it’s very good, yeah. How different people view things and how they prepare for a concert and what kind of emotions they go through and stuff, it helps you to, you know, see your weaknesses and strengths and what you want to do next (Emma).

### Structures (Essential Features) of the Lifeworld

The themes of “temporality” and “intersubjectivity” were evident throughout musicians’ narratives. Temporality refers to lived time as it is perceived and experienced whereas intersubjectivity (or sociality) refers to the experience of relationships with other people (Ashworth, 2016).

#### Temporality

For all participants, time was an important element. They spoke about the lack of time and how it negatively affected the development of their performing skills:

(…) there’s finding that we are very, very time poor at the moment in our lives. And that’s something that I very much regret. So, I very much want to do that before I feel that I’m completely over the hill [laughs], as far as my own playing is concerned, so I’m desperately trying to hang on to the skills that I have (…) (Tom).

A lot of the concerts that I do involved organizing it [them] (…) And that gets me down sometimes because occasionally you find (…) that (…) you get to the rehearsal space or the performance space and you think “oh, bloody hell, I’ve got to play my saxophone as well!.” You’ve completely forgotten in the millstream of all the organizing that you’re actually involved… and then I feel guilty and a bit angry that the whole reason for doing this all is to play the saxophone basically, and then in order to make it happen, I’ve had to kind of put to one side my own sort of personal practice (Robert).

I don’t get to practise an awful lot. I love to play at the moment, but I don’t find much free time to play, because very often if I go to the piano, the kids will come and sit on my feet, or they’ll insist that I start playing “Thunderbirds”’ instead or something else, you know, the “Bob The Builder” theme tune… (Kelly).

When you don’t have that much time (…), I always feel like I’ve kind of short-changed the music (Emma).

#### Intersubjectivity

Descriptions of relationships with fellow musicians were evident in all interviews. Tom and Robert defined themselves through relationships with other musicians and expressed a strong sense of belonging to a community. This sense of community mediated performance anxiety and featured prominently in positive musical experiences:

I feel the most comfortable with orchestral music, so really you’re talking of my musical highs have been within an orchestra. So, concerts to me have been wonderful orchestral concerts where I felt I that I’ve risen to the occasion or the challenge that that I felt that I played my part as a member of a team (Tom).

I think that way of being a musician, who you’re not the lone virtuoso musician, sort of out there defining themselves through that, you’re actually part of a group of musicians and you define yourself through the musical relationships that you have, but—at the same time—you still have a vision (Robert).

For Kelly, band rehearsals involved a social component that facilitated cohesion and a shared vision in the band:

… we’d all get together, we’d probably chat for a bit, probably have a cup of tea, we’d talk about all sorts of unrelated things and then we’d play and try some things and discard some things that weren’t working, or try pieces in different ways to find good groove or good… you know, a good feel (Kelly).

Emma felt that talking with other musicians was very helpful:

Particularly talking about things with others is very-very useful, actually. Erm… recently that’s been, kind of, more helpful than the actual lessons I had, really (Emma).

## Discussion

The present findings suggest that professional musicians’ experience of performance anxiety is multifaceted. The lived experience of performance anxiety for the four musicians interviewed was characterized by both a physical and a psychological dimension. Anxiety was an intensely felt emotion that was depicted through descriptions of physical pain and humiliation, and that was closely related to perceived lack of confidence and the pressure of feeling a need to compete with other musicians. The experience of anxiety was lived through the body. The centrality of the body in this experience is analogous to the descriptions of anger depicted in Eatough and Smith (2006), suggesting that intense emotions are experienced from within the body. This could relate to perceptions of the increased physiological activity that has been found to correlate with performance anxiety in experimental studies, such as Yoshie et al. (2009) and Gomez et al., 2018 who reported that MPA has specific effects on hypothalamic-pituitary-adrenal axis, autonomic nervous system, and cognitive activity. The pressure of competition when working in environments where there is social evaluative threat and its contribution to performance anxiety has previously been highlighted by Kenny et al. (2004) and Yoshie et al. (2009). For these four musicians, performance anxiety had strong negative connotations. Its most profound negative effects were a decrease in the perceived quality of playing, thus reducing their enjoyment of playing and a sense of preventing these musicians from reaching their full potential. This finding lends support to studies reporting maladaptive effects of MPA, such as Burin and Osorio (2017), Yoshie et al. (2009), and Osborne and Kenny (2008). Negative descriptions of performance anxiety also focused on how the experience produced negative feelings toward themselves, a finding which may relate to the function of music as a determinant of self-concept in professional musicians (Burland and Davidson, 2002). However, one participant had reconceptualized MPA into being a sign of excitement and motivation, thus focusing on its adaptive function. Research on adult musicians has previously acknowledged these different perceptions in musicians (see, e.g., Papageorgi et al., 2013; Papageorgi and Welch, 2014).

The development of coping strategies was instrumental in their sense of being able to moderate or deal effectively with the demands of performance and to keep anxiety under control. Musicians’ narratives suggested that coping with anxiety involved practical strategies focusing on addressing the problem (problem focused) and strategies that aimed at eliminating the negative emotion (emotion focused). This indicates that performance anxiety was conceptualized both in practical and in emotional terms. Burland and Davidson (2002) also found that in dealing with the demands of performance, musicians used both physical measures and more internal responses. The musicians mostly referred to self-help strategies and use of personal resources in dealing with anxiety. With the exception of one participant, musicians did not mention specialized (medical or psychological) treatments, a finding in line with earlier studies (e.g., Kenny et al., 2004; Ryan and Andrews, 2009). One reason may be that musicians often interpret the symptoms of MPA as being normal and inherent to the profession, and so do not seek treatment (Burin and Osorio, 2017). Maintaining a realistic outlook to the performance also seemed to reduce the pressure of performance, a strategy previously described as a mediating factor for performance anxiety (Steptoe and Fidler, 1987). It was somewhat alarming that musicians reported to rely mostly on self-help strategies and personal resources and did not seek professional help to overcome MPA. Higher education curricula should allocate resources to the development of coping skills in performers in training to facilitate healthy emotional and physical skills development. These coping skills may relate to performance management (such as coping with performance anxiety, improving stamina, managing every day stress), the improvement of technical preparation skills (such as maximizing the quality of practice, maintaining perseverance and motivation), as well as facilitating professional development (such as organization, general musical knowledge and networking skills) (Papageorgi et al., 2010b). Another important skill is the ability to dissociate from the work environment, which could function as a form of protection against the high demands of the performing profession (Hildebrandt et al., 2012). Musicians should also be informed about the various treatments available (e.g., behavioral interventions, cognitive interventions, cognitive-behavioral therapy, combination of treatments; for an overview see Kenny, 2005), and that support by registered professionals can be beneficial in coping with performance anxiety and reducing the negative effects it can have on performance quality. Evidence reviewed by Kenny (2005) suggest that behavior rehearsal, cognitive restructuring, combined self-instruction and progressive muscle relaxation and combined self-instruction and attentional training can improve the quality of performance. Psychoeducational workshops have been shown to reduce self-reported MPA and increase performance quality in adult musicians (Hoffman and Hanrahan, 2012) and such initiatives should constitute regular components of higher education curricula for performers.

Overcoming performance anxiety was critical for musicians’ careers, which came with professional maturity and becoming more pragmatic. A negative association between experience and MPA has been previously reported by Papageorgi et al. (2013) and Ryan and Andrews (2009). A sense of belonging to a supportive community and successful communication with fellow musicians was considered very important, as also reported elsewhere in the literature (e.g., Papageorgi et al., 2010a). Burland and Davidson (2002) reported that their professional musicians tended to perceive interactions with peers as opportunities for learning, inspiration and motivation. Supportive relationships may, therefore, act as a moderating factor in dealing successfully with performance anxiety, corroborating Papageorgi et al. (2010b), who highlighted the importance of peer support in coping with the demands of performance. Schneider and Chesky (2011) also found that college music students with less frequent experiences of performance anxiety reported greater perceived social support. Recently, Zarza-Alzugaray et al. (2020) provided further evidence for the importance of social support (by family, teachers and peers) in the development of performance self-efficacy and the mediating role of MPA in the process, and pointed to the necessity of establishing true social support that acts as a motivator to face performance challenges.

## Conclusion

A phenomenological perspective applied to the study of performance anxiety has contributed to our understanding of this experience. Through IPA of participant professional musicians’ interviews, it became evident that performance anxiety is experienced intensely by musicians and that it can have profound effects, usually negative. The lived experience of performance anxiety is multifaceted, characterized by a physical and a psychological dimension. Findings also highlighted the inherently occurring need to resort to constructive personal strategies for dealing with the demands of performance. We suggest that IPA is very promising in further illuminating the lived experience of performance anxiety.

One caveat is that it is possible that the participants may have considered the interviewer as someone who shared similar concerns (being a performer), but it is equally possible that they perceived the interviewer as an outsider and were cautious in their narratives due to an inherent power relationship between researcher/participant. Furthermore, it is important to note that this study focused on musicians who performed as part of a group (orchestra, band), and so did not look at soloist musicians who may well have different (more intense) experiences, judging by the previous literature on the increased sense of MPA in solo performance (Papageorgi et al., 2007, 2013; Ryan and Andrews, 2009; Burin and Osorio, 2017; Coskun-Senturk and Cırakoglu, 2018). Consequently, future research would benefit from looking at the performance anxiety experiences of solo musicians, as well as investigating the lived experience of anxiety as described by musicians who are at different stages in their musical development, including amateur as well as professional musicians. We suggest that IPA is a useful research tool that can facilitate our understanding of the subjective experience of performance anxiety (how it is felt and understood at an individual level) and can thus be useful in the sensitizing policy in the development of tailor-made intervention programs for musicians.

## Data Availability Statement

The raw data supporting the conclusions of this article will be made available by the authors upon request, without undue reservation.

## Ethics Statement

The studies involving human participants were reviewed and approved by the Institute of Education Ethics Committee and according to the expectations of the ESRC, BERA, and BPS. The patients/participants provided their written informed consent to participate in this study.

## Author Contributions

Both authors contributed extensively to the work presented in the manuscript. Both authors contributed to the article and approved the submitted version.

## Conflict of Interest

The authors declare that the research was conducted in the absence of any commercial or financial relationships that could be construed as a potential conflict of interest.

## Footnotes

1 Please note that names of participants have been changed to protect confidentiality.
